# Assessing the Formation of Experience-Based Gender Expectations in an Implicit Learning Scenario

**DOI:** 10.3389/fpsyg.2017.01485

**Published:** 2017-09-07

**Authors:** Anton Öttl, Dawn M. Behne

**Affiliations:** Speech Lab, Department of Psychology, Norwegian University of Science and Technology Trondheim, Norway

**Keywords:** implicit learning, artificial language, frequencies of exposure, visual world eyetracking, gender representations, categorization, experience-based probabilities

## Abstract

The present study investigates the formation of new word-referent associations in an implicit learning scenario, using a gender-coded artificial language with spoken words and visual referents. Previous research has shown that when participants are explicitly instructed about the gender-coding system underlying an artificial lexicon, they monitor the frequency of exposure to male vs. female referents within this lexicon, and subsequently use this probabilistic information to predict the gender of an upcoming referent. In an explicit learning scenario, the auditory and visual gender cues are necessarily highlighted prior to acqusition, and the effects previously observed may therefore depend on participants' overt awareness of these cues. To assess whether the formation of experience-based expectations is dependent on explicit awareness of the underlying coding system, we present data from an experiment in which gender-coding was acquired implicitly, thereby reducing the likelihood that visual and auditory gender cues are used strategically during acquisition. Results show that even if the gender coding system was not perfectly mastered (as reflected in the number of gender coding errors), participants develop frequency based expectations comparable to those previously observed in an explicit learning scenario. In line with previous findings, participants are quicker at recognizing a referent whose gender is consistent with an induced expectation than one whose gender is inconsistent with an induced expectation. At the same time however, eyetracking data suggest that these expectations may surface earlier in an implicit learning scenario. These findings suggest that experience-based expectations are robust against manner of acquisition, and contribute to understanding why similar expectations observed in the activation of stereotypes during the processing of natural language stimuli are difficult or impossible to suppress.

## 1. Introduction

When processing a word referring to a human being, we typically activate an expectation as to whether it refers to a female or a male person. For most, the word “*nurse”* likely triggers a female representation, whereas “*mechanic”* likely triggers a male representation (see Misersky et al., 2013 for a survey of estimated gender distributions across several languages). While such expectations can be traced to societal stereotypes and also to actual gender distributions (Gygax et al., 2016), certain languages additionally provide grammatical gender cues that may or may not be consistent with stereotypical information. For example, in Spanish grammatical gender is typically marked by the determiner “*el”* (masculine) or “*la”* (feminine), as well as by means of suffixation, as in “*camarer*o” (male waiter) vs. “*camarer*a” (female waiter). Though grammatical cues in principle could override stereotypical information, previous research has shown to the contrary that stereotypical gender information is activated automatically, even in cases where it is not needed for discourse coherence (Pyykkönen et al., 2010). One challenge to examining the interplay between linguistic and experience-based sources of gender information at the lexical level is the complexity of gender coding systems found in natural languages, as well as the stereotypes associated with them. The present study therefore employs an artificial language paradigm, in which aspects of interest, gender-coding and experience-based expectations, can be simulated in a laboratory setting and studied in isolation. While previous research has shown that an artificial language paradigm is adequate for studying the formation of new representations (Magnuson et al., 2003) and also more specifically the emergence of probabilistic gender expectations (Öttl and Behne, 2016), the aim of the present study is to investigate whether mode of acquisition affects the formation of new representations. To achieve this aim, the present study replicates an experiment in which experience-based gender expectations were induced in an explicit learning situation, but shifting the context to an implicit learning scenario.

One reason why mode of acquisition might be expected to affect how new representations are processed or stored is based on the assumption that mode of acquisition guides a learner's attention (e.g., Marsden et al., 2013). For example, prior knowledge that a to-be-acquired artificial lexicon encodes referential gender by means of suffixation likely makes a learner consciously focus on the suffixes available in the language, but also on visual features that are likely to be informative of gender in possible referents. Thus, one might expect that in an explicit learning scenario, attention both to relevant linguistic and visual information would be enhanced from the onset of learning. If, on the other hand, a learner lacks explicit knowledge about the structure underlying the artificial language, detecting and correlating the linguistic and visual regularities necessarily requires at least one additional learning step. In the latter case, relevant gender information could potentially remain undetected in one or both modalities, resulting in less attention to gender, which again could have implications for the associations that are being established in the learning process. Crucially, whether implicit learning results in conscious or unconscious knowledge about the gender coding system, a learner may successfully acquire unanalyzed mappings between words and referents, i.e., forming an association between two holistic units (word and referent) without realizing that there is a systematic relationship between suffix and visual gender cues. Thus, in terms of mastering word-referent associations, similar learning outcomes are possible from both modes of acquisition, but superficial similarities may also overshadow potential differences in processing, such as in the formation of experience-based expectations.

In a recent study in which participants acquired a gender-coded artificial language based on a suffixation system similar to that of Spanish, Öttl and Behne (2016) found that experience-based gender expectations (1) can be simulated by manipulating relative frequencies of exposure to male vs. female referents during training, (2) surface during online lexical processing, and (3) are not overridden by linguistic cues. In this study, participants acquired associations between spoken pseudowords on the one hand and visual referents on the other. Whereas pseudowords were marked for gender by means of suffixation, visual referents represented novel imaginary figures whose facial features were gendered by means of stereotypically masculine or feminine traits, making gender an integral feature of the referents. To induce experience-based expectations about a referent's gender, relative frequencies of exposure to male vs. female words and their associated referents were manipulated during training. Thus, each wordstem would be unambiguously associated with a figure's overall features (color, texture, and shape), and would also be more likely to appear with either the female or the male suffix (or both would be equally likely) and the figure of the corresponding gender. Results showed that participants were faster at identifying those referents that were consistent with the induced expectation than those that were not. This finding indicates that that learners did not solely rely on the unambiguous information that was available from the linguistic input, but also developed experience based expectations about referential gender that surfaced during subsequent processing. While this study provides insights into the formation and activation of experience-based gender expectations on the lexical level, it was based on an explicit learning situation in which learners were initially informed about the gender coding system underlying the materials to be acquired, and it is not clear to which extent the observed effects depended on this prior knowledge. The present study replicates this experiment in order to assess whether experience based gender expectations can also be induced in an implicit learning scenario, and thereby provides a more stringent simulation, as the category of interest (gender) is not highlighted to the learners. Since overt attention to gender is likely to be lower in an implicit learning situation, one possibility is that gender expectations are not induced. A less obvious possibility is that implicit learning results in stronger gender expectations. The rationale underlying the latter possibility is that in an explicit learning scenario, learners know that the information that is needed to identify the gender of a referent is encoded in the suffix, and they may therefore be less sensitive to probabilistic information that potentially contradicts the linguistic information.

Both in the original experiment and the current replication, participants acquire word-referent associations by immediate feedback. Images of four possible referents are presented on a screen, a pseudoword is presented auditorily, and participants have to select one of the available candidates. Of particular importance for the current experiment, other lines of research have demonstrated that learners may use cross-situational statistics to detect word-referent mappings, i.e., tracking the co-occurrence of potential referents for a given word across trials can be sufficient to establish the correct mappings over time, even in the absence of direct evidence, see e.g., Yu and Smith (2007). In addition to the direct mapping that is enabled by immediate feedback, learners may also apply additional learning mechanisms. For example, as participants become increasingly familiar with the stimuli, recognizing one or more of the distractors means that these can be eliminated from the possible candidates (for a discussion of the mutual exclusivity principle, see Markman and Wachtel, 1988). Crucially, while word-referent associations may be acquired as one-to-one mappings, the structure of the artificial language implies that parallel mappings exist between word stems and figures on the one hand, and between suffixes and gender on the other, whether the participant becomes consciously aware of this or not. If, and when, these component-based mappings are detected, they potentially provide learners with top-down information that can boost acquisition. The present study does not investigate these learning mechanisms *per se*, but see e.g., Koehne and Crocker (2014) for a recent study on the interplay between such learning mechanisms.

If experience-based expectations can be detected in an implicit learning scenario, we expect participants to be quicker at recognizing referents whose gender is consistent with an induced expectation relative to referents whose gender is inconsistent with an induced expectation. Additionally, if such effects are replicated, any observed differences in strength will inform how mode of acquisition affects the resulting representation. One possibility is that stronger gender expectations are observed in an implicit learning scenario relative to an explicit learning scenario. This finding would suggest that in an explicit learning scenario, unambiguous linguistic information attenuates the impact of experience based information, even if it does not override it *per se*. On the other hand, finding weaker gender expectations in an implicit learning scenario would suggest that the explicit instructions, at least to some extent, contributed to the development of gender expectations, most likely by priming learners' awareness of gender cues in both modalities. These questions have implications for understanding the impact of different modes of acquisition, but also the cognitive representation of gender information, by assessing the impact of explicit awareness of gender coding on the resulting expectations.

## 2. Methods

### 2.1. Design

The present experiment is a replication of an experiment reported in Öttl and Behne (2016), where participants were trained in a gender-coded artificial language in which the frequency of exposure to different words and referents were manipulated to induce experience-based expectations. In the current replication, one aspect of the original experiment was modified. Rather than explicitly informing participants that they would acquire a gender-coded language, this information was withheld in order to establish an implicit learning situation. Apart from this modification and the removal of one example slide illustrating the gender coding, the two experiments are identical, and consist of three parts: (a) a pre-test in which participants are familiarized with the stimuli to be acquired, (b) a training phase in which participants learn new word-referent associations, and (c) a post-test in which the processing of the newly acquired representations is evaluated. The different parts of the experiment, and the frequency manipulation, are outlined in more detail in Section 2.4.

### 2.2. Participants

Twenty native speakers of Norwegian (10 male, mean age = 23.1, *SD* = 2.8) were recruited at the Norwegian University of Science and Technology (NTNU) in Trondheim. All participants reported normal hearing and normal or corrected-to-normal vision and were compensated for participation with a gift certificate, and gave their informed consent by signing a form that had been approved by the Data Protection Official for Research at Norwegian Universities (NSD).

### 2.3. Materials

All materials used in the experiment are identical to those used in Öttl and Behne (2016), where the development of the stimuli is described in more detail.

#### 2.3.1. Auditory stimuli

**Figure 1:**
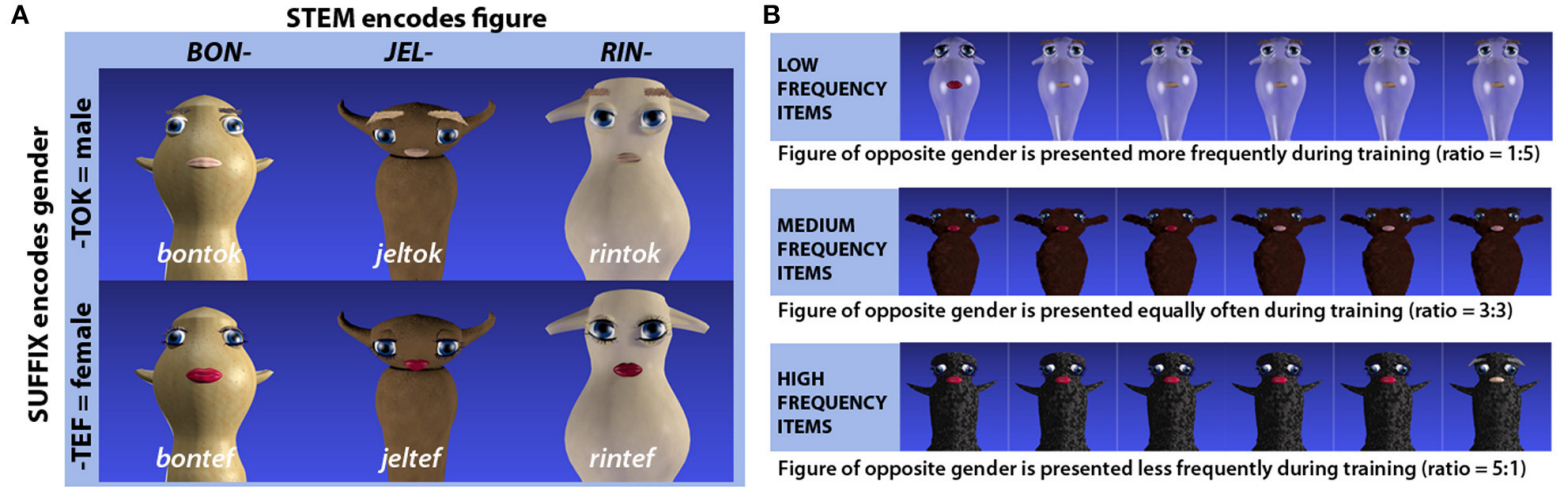
**(A)** Schematic representation of the structure underlying the pseudowords and how stems and suffixes relate to different visual features (character and gender respectively) of the referents. Three out of 12 word stems and character pairs are exemplified here. **(B)** Example of how presentation frequencies for male vs. female versions of the same imaginary figures were used to induce biased gender likelihoods. Originally published in Öttl and Behne (2016).

The artificial lexicon was designed to encode gender through suffixation and consisted of 24 pseudowords. These were made up of 12 pseudoword stems, which were paired with two different pseudosuffixes (“*-tef”* and “*-tok”*) (see Figure 1). Structurally, the pseudowords were made up of two syllables, each of which consisted of a consonant-vowel-consonant sequence. The audio recordings of the pseudowords were spoken by a young adult female native speaker of Urban East Norwegian (Kristoffersen, 2000) and recorded with a Røde NT1-A microphone at a sampling rate of 44.1 kHz in Praat version 5.3 (Boersma and Weenink, 2017). As fine acoustic-phonetic detail can be actively used to predict upcoming information during online processing at the lexical level (Salverda et al., 2003), participants could theoretically exploit acoustic-phonetic cues from the word stem to predict whether it would end in “*-tef”* or “*-tok.”* To ensure that the suffix could not be predicted from the stem, the 24 original recorded tokens were therefore cross-spliced, i.e., audiofiles (e.g., “*bontok”* and “*bontef”*) were cut at the syllable boundary to obtain separate audiofiles for stems and suffixes (e.g., “*bon”*^*a*^,“*tok”*^*a*^,“*bon”*^*b*^,“*tef”*^*b*^) which were then recombined to produce additional tokens (e.g., “*bon*^*a*^*tef*^*b*^”,“*bon*^*b*^*tok*^*a*^”) in Praat (Boersma and Weenink, 2017) that were used interchangeably throughout the experiment. The average duration of pseudowords was 865 ms (*SD* = 71). The timepoint at which gender information become available can be identified as the onset of the vowel in the second syllable. Both for words ending in *-tef* and words ending in *-tok* this occurred 440 ms after word onset (*SD* = 46 and *SD* = 47 respectively).

#### 2.3.2. Visual stimuli

Imaginary figures were designed to provide referents for the artificial language outlined above, and could be either male or female. This image set was entirely symmetric in the sense that it was based on 12 base figures without any cues to gender. These base figures were distinguished in terms of overall shape, color and surface texture (e.g., shiny, furry, matte), and for each base figure, a male and a female version was created. While female figures had red lips and long eye-lashes, male figures had lighter but short eye-lashes, slightly smaller pink lips and bushy eyebrows. All gender cues were thus local features of the facial region, in contrast to the global features distinguishing the different base figures from each other. Images were created using Blender 3D modeling software, version 2.60 (Blender Foundation, 2012).

#### 2.3.3. Sound–image associations

Each of the 12 word stems was consistently linked to one of the 12 base figures, while the two suffixes were consistently linked to the gender identity of a given figure (see Figure 1). The links between word stems and base figures were randomly assigned for different participants, and for one half of the participants, the suffix “*-tok”* was assigned to male and “*-tef”* to female figures, while for the other half, the gender assignment was reversed.

### 2.4. Procedure

Testing took place in a sound attenuated booth in the Speech Lab at the Department of Psychology at NTNU. Participants were seated approximately 70 cm from a computer display. Eprime 2.0.8.90 was used to run the experiment and a SmartEye 5.8 remote system was used for the collection of gaze data (at a sampling frequency of 60 Hz), with SmartEye extension for Eprime (Version 1.0.1.49) to handle the communication between the two. Auditory stimuli were presented over AKG MKII K271 headphones and responses were collected using a computer mouse connected to the stimulus PC. The experiment was controlled from outside the booth.

Testing consisted of a pre-test (24 trials), five training blocks (72 trials each) and a post-test (144 trials), and the experiment duration was approximately 1 h, including an additional 15–30 min for calibration, questionnaires and debriefing. Participants were informed about the overall structure of the experiment (i.e., that it contained a pre-test, training blocks and a post-test) prior to participation, but were naïve to critical aspects of the experiment (i.e., to the gender coding and the frequency manipulation).

#### 2.4.1. Pre-test

Participants were informed that they would be familiarized with the words and images that they would acquire in the course of the experiment, that they would see four characters on the screen, listen to a nonsense word, and then have to guess which of the images the word belonged to by clicking with the mouse on one of the images. Each trial began with a gaze contingent fixation cross in the center of the display. As soon as this had been fixated for 500 ms, four images (two male and two female figures) appeared on the display. Five hundred ms later, a pseudoword corresponding to one of these was presented over the headphones. Once a response had been made, a gray frame appeared around the selected image to indicate that the response had been registered. Five hundred ms later, all images were removed from the display. If no image was selected within 4,500 ms, the experiment would automatically move on to the next trial. Each of the 24 stimuli appeared once as a target and three times as distractor. Image displays were randomized, but never featured the male and the female version of the same base figure at the same time. Nor would the same word stem appear as a target in two consecutive trials. These constraints on the randomizations were implemented in order to make it more difficult for participant to detect the structure of the materials. At the end of the pre-test participants received feedback as to how many percent of their answers were correct, and were informed that they would now proceed to the training part.

#### 2.4.2. Training blocks:

Participants were told that the task would be the same as in the pre-test, but that they would receive feedback after each response whether they had selected the correct image or not. As soon as a participant had selected one of the images, this would receive a green frame if the response was correct, or a red frame if the response was incorrect. Five hundred ms later (or 4,500 ms after word onset, if no image had been selected), the incorrect images were removed from the display, while the correct image remained until the pseudoword had been repeated over the headphones. Randomization procedures were identical to the pre-test. Presentation frequencies to male vs. female realizations of the same base figures (and correspondingly the associated stem-suffix combination) were manipulated to create three different frequency groups. For one third of the image pairs, the ratio of presentation was 1:5 for male vs. female versions, resulting in the male version becoming a low frequency item and the female version a high frequency item. For another third of the items, the presentation ratio was reversed to a presentation ratio of 5:1 for male vs. female realizations. For the final third, male and female realizations were presented equally often (medium frequency items). Each training block was followed by feedback on the percent of the responses which were correct, and a 30-s break.

#### 2.4.3. Post-test:

**Figure 2:**
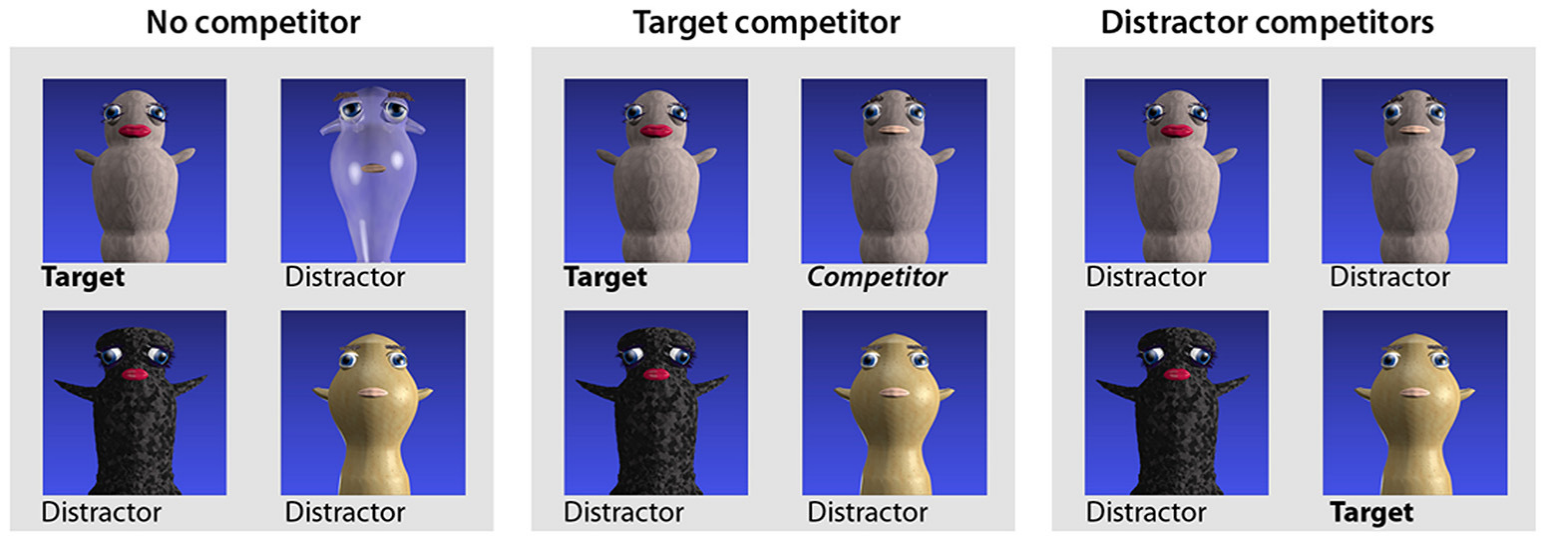
Different trial types used in the post-test. Originally published in Öttl and Behne (2016).

The trial structure and randomization procedures were identical to the pre-test. With respect to the visual displays the post-test differed from both the pre-test and the training blocks in that three different trial types (within participants) were used to investigate different aspects of processing (Figure 2). One trial type, referred to as *no competitor trials*, was identical to the pre-test, and always contained four unrelated images. In these trials, any target image could unambiguously be identified by the word stem and the global features alone (e.g., a target image associated with the word “*gontef”* would be accompanied by three distractor images associated with unrelated words “*sjestok,” “kestef,”* and “*lentok,”* rendering both the suffix and the visual gender cues redundant). A second trial type referred to as *target competitor trials*, contained an image associated with the same base figure as the target word, but of the opposite gender. For example, based on Figure 2, if the target word was “*gontef,”* the image associated with “*gontok”* would be among the distractors, and the target and competitor would be distinguishable only by the suffix and the local gender features. Finally the third trial type, *distractor competitor trials*, featured two distractors constituting an image pair. The latter trial type was included to prevent participants from adopting a response strategy according to which the mere presence of an image pair would indicate that the target was among the pair. In the post-test, all words/figures appeared twice as a target in each of the three trial types, regardless of presentation frequencies during training.

Based on pre-testing and the results reported in Öttl and Behne (2016), we expect participants to acquire the 24 words within the training blocks provided. If participants also successfully acquire the gender-coding underlying the stimulus materials, post-test scores for target-competitor trials (that require explicit gender identification) should be similar to no-competitor and distractor-competitor trials. Regarding response times for successfully acquired word-referent pairs, we expect participants to be quicker at recognizing high-frequency items than low-frequency items, provided that gender information is readily available. Crucially, we also expect the gazedata to provide information on how quickly gender information becomes available.

#### 2.4.4. Statistical procedures

All analyses are based on linear mixed effects models in R, version 3.0.2 (R Core Team, 2013), using the *lmer* and *glmer* functions (depending on the dependent variable being continuous or binomial) from the lme4 package (Bates et al., 2014). Model comparisons were performed using log likelihood tests, using a forward-testing approach: fixed effects are included one at a time, and their contribution to improving model fit is evaluated by comparing the respective model to one that is identical except for not containing the fixed effect in question. Model comparisons to arrive at the best fitting model are included in the Supplementary Materials. In line with current recommendations (Barr et al., 2013), maximal random effects structure was used, i.e., in addition to random by-subject intercepts, random by-subject slopes were included for the fixed effects being tested. Also, the contribution of random slopes to the model fit was assessed using the same forward testing approach as described above. The inclusion of random slopes was warranted for all models. To obtain *p*-values for the best fitting models, lme4 was used in conjunction with the lmerTest package (Kuznetsova et al., 2014).

When trial type is included as a fixed effect in a model, the intercept represents trials where no image pairs were present, and this estimate can be directly compared to the adjustments required for target competitor trials and distractor competitor trials. Correspondingly, when frequency is included as a fixed effect, the intercept represents low frequency items and direct comparisons to medium and high frequency items are available from the model estimates. When both effects are included in the same model, the intercept represents the estimate for low frequency items in no competitor trials. To facilitate interpretation in cases where an interaction is not included in the model, the relevant estimates for the given factor are reported in isolation, using proportions instead of log likelihoods and milliseconds instead of log transformed milliseconds. To test differences between the factor levels that cannot be read directly from the model (i.e., between medium and high frequency items or between target competitor and distractor competitor trials), the factor levels were reordered prior to recalculating the same model, in line with recommendations outlined in Singer and Willett (2003).

## 3. Results and discussion

Section 3.1 below presents the learning curves to evaluate participants' overall performance throughout the training. Sections 3.2 through 3.4 focus on results from the post-test only: accuracy data (3.2), response times (3.3), and gazedata (3.4). Interim discussions are included in the respective sections.

### 3.1. Learning curves

**Figure 3:**
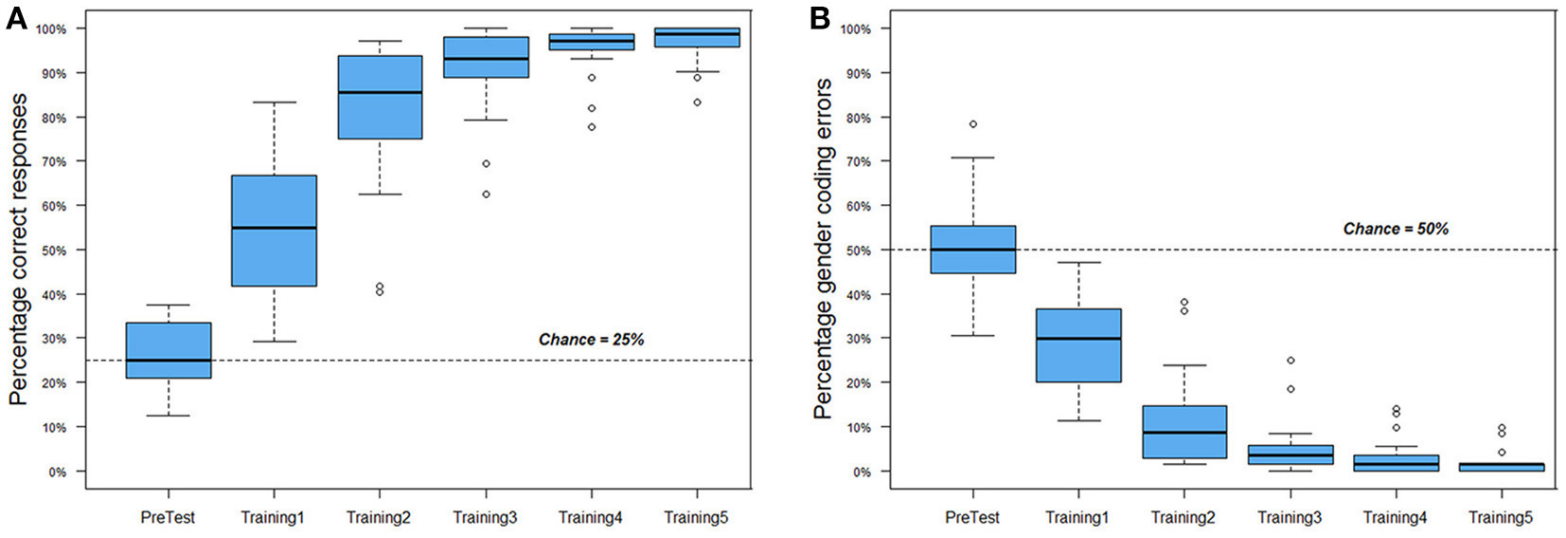
Performance during traing blocks. Boxes represent median values and upper and lower quartiles. **(A)** Overall accuracy. **(B)** Gender-coding errors.

As shown in Figure 3A, participants started at chance levels in the pre-test, and successfully acquired the artificial language within the provided training blocks. In the last training block (Training5), only two participants scored below 90% correct (at 88.9 and 83.3%). Since they demonstrate a similar progression in learning as the other participants, they are included for further analysis.

Figure 3B presents the proportion of gender-coding errors. During training, a gender-coding error indicates that a participant in addition to not mastering stem-character links fails to acknowledge suffix-gender links. A low proportion of gender-coding errors would indicate that the gender-coding system had been deciphered. Figure 3B shows that participants did not immediately detect the gender-coding. That these errors decrease in parallel with overall learning increases implies that participants could in principle be forming instance-based mappings. If this were the case, we would not expect any learning transfer to occur between the two versions of a given character type, as these would be treated as independent instances. To follow up on this possibility, participants' mean accuracy at the second exposure to a given character type in the first training block was analyzed. When this second exposure constituted a true repetition (e.g., the exposure to “*bontef”* had been preceded by one exposure to “*bontef”* earlier in the training), participants scored at 44% correct (*SD* = 18.7). When the exposure constituted a false repetition (i.e., the exposure to “*bontef”* had been preceded by exposure to “*bontok,”* but “*bontef”* had not yet been encountered in training) participants scored at 48% correct (*SD* = 26.6). The difference between the two scores was not significant [*t*(19) = 0.499, *p* = 0.623]. Since participants are also likely to use cross-situational information (as discussed in the introduction), and the randomization of trials did not control for the time-point at which these second exposures constituted true or false repetitions, a parallel analysis of trial numbers was conducted. The average trial number was 19 (*SD* = 3) for true repetitions and 21 (*SD* = 7) for false repetitions, but this difference was not significant [*t*(19) = 1.156, *p* = 0.262]. Taken together, these results suggest that participants needed some time to detect the gender-coding system, but that they nevertheless were sensitive to the referential overlap between male and female versions of a character early on. This is consistent with research demonstrating that learners can track one-to-one and one-to-many mappings in parallel, particularly when it comes to natural categories (Gangwani et al., 2010).

### 3.2. Post-test accuracy

As outlined in Section 2.4, the post-test includes target competitor trials, where participants are required to actively distinguish the male and the female realization of the same character. An elevated error rate in these trials would suggest that participants primarily relied on information from the word stem, and therefore likely experienced targets and competitors as ambiguous referents. An alternative scenario would be that a decrease in performance is indicative of switching costs or more general confusion or surprise at the new trial type. Importantly, the performance in distractor competitor trials is likely to be informative, as these are visually identical to the target competitor trials, however without requiring a gender distinction to be made. Potentially, the errors committed in target competitor trials may also reflect effects of frequency, in which case accuracy is expected to be highest for high frequency items and/or lowest for low frequency items.

To analyze the effects of trial type and frequency on accuracy, a binomial linear mixed effects analysis was performed. Including a fixed effect for trial type (model A) led to a significant improvement over the null model [χ2(2) = 7.8, *p* < 0.05]. To assess the effect of frequency, two additional models were tested: one in which a fixed effect for frequency was added to model A (model B), and one that also included its interaction term with trial type (model C). Neither of these led to significant improvements over model A [model B: χ2(2) = 2.0, *p* = 0.367], [model C: χ2(6) = 7.59, *p* = 0.270].

Fixed effects estimates from model A, which provided the best fit for the data, are presented in Table 1. For trials where no image pair was present, model A estimates a score of 98.4% correct (95% CI [97.1, 99.1]). In trials where a distractor competitor pair was present, this score is estimated to be 96.5%, which is significantly lower (95% CI [95.0, 97.6], *p* < 0.01). Also in trials where a target competitor was present, performance is significantly worse at 94.6% correct (95% CI [88.8, 97.5], *p* < 0.001). No significant difference was found between target and distractor competitor trials (*p* = 0.229). The lower accuracy in target competitor trials suggests that the gender-coding did lead to some difficulties. However, since accuracy was also lower in distractor competitor trials, the decrease in performance cannot be fully attributed to difficulties with gender-coding, but at least partially to the new visual displays. That no effects of frequency were observed may be due to ceiling effects.

**Table 1:** Model estimates for accuracy data (Day 1).

**Fixed effects**	**Estimate**	**Std. Error**	***z*-value**	**Pr(<z)**
(Intercept)	4.089	0.291	14.05	<0.001
**TRIAL TYPE**				
Distractor competitors	−0.761	0.279	−2.73	<0.01
Target competitor	−1.218	0.354	−3.44	<0.001

### 3.3. Response times

In the following, only correct responses that were longer than 300 ms are analyzed (95% of the data), as earlier responses are more likely to be erroneous button presses than to reflect actual recognition (e.g., Baayen, 2008). As suggested in Baayen (2008), response times were log transformed prior to the analysis. As shown in Section 3.2, performance differed between the trial types, and this implies that removing incorrect responses affected the three trial types to different degrees: 97% of the data were analyzed for no competitor trials, 96% for distractor competitor trials, and 85% for target competitor trials.

**Figure 4:**
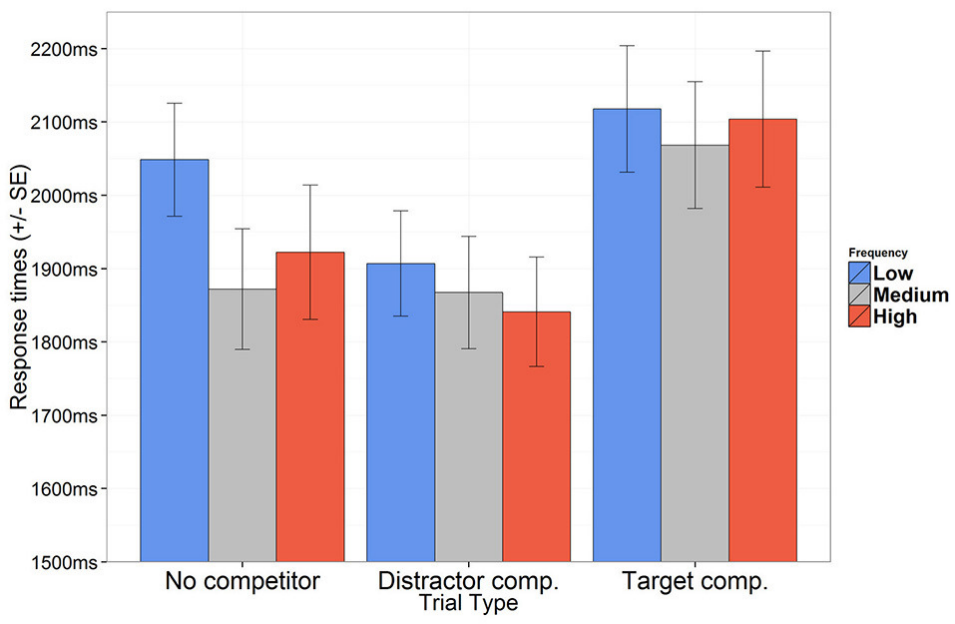
Mean response times for correct responses according to trial type and presentation frequencies. Error bars represent SE.

Based on the findings in Öttl and Behne (2016), response times are expected to be longer for trials where a target competitor is present, compared to trials where none is present (partly because participants need to await auditory information from the suffix in order to identify the target). For trials where a distractor competitor pair is present, response times are expected to be shorter, since two response alternatives can be eliminated as soon as the stem has been identified. If experience based expectations can be replicated in an implicit learning situation, these are expected to surface as longer response times for low frequency items and/or shorter response times for high frequency items, reflecting relative ease of processing. The best fitting model includes fixed effects for trial type and frequency, but not their interaction term. The estimates obtained from this model are summarized in Table 2, and the aggregated data are presented in Figure 4.

**Table 2:** Model estimates for response time data.

	**Estimate**	**S.E**	**df**	***t*-value**	**Pr(>t)**
(Intercept)	7.543	0.037	19.1	203.84	<0.001
**TRIAL TYPE**					
Distractor comp.	−0.035	0.015	74.5	−2.43	<0.05
Target comp.	0.076	0.022	19.1	3.45	<0.01
**FREQUENCY**					
Medium	−0.050	0.019	23.0	−2.67	<0.05
High	−0.039	0.015	57.1	−2.51	<0.05

The response time is estimated at 1,831 ms (95% CI[1,699, 1,974]) when no competitor is present. Relative to this, response times were significantly longer in target competitor trials (1,976 ms, 95% CI[1,835, 2,127]), and significantly shorter in distractor competitor trials (1,768 ms, 95% CI[1,645, 1,900]). Compared to the overall response time for low frequency items (1,908 ms, 95% CI[1,782, 2,042]), response times were significantly shorter both for medium frequency items (1,814 ms, 95% CI[1,683, 1,955]) and high frequency items (1,836 ms, 95% CI[1,698, 1,984]). These results show that experience based expectations reported in Öttl and Behne (2016) were successfully replicated.

### 3.4. Gazedata

Gazedata were collected during the entire post-test, and provide a continuous record of which of the four images in the display was fixated during each trial. Each obtained gaze coordinate was classified as pertaining to one of four regions of interest (corresponding to the four image positions on the display), or as falling outside these regions. To compensate the 200 ms that are typically estimated for the planning and execution of eye movements (e.g., Matin et al., 1993), the time windows of analysis are shifted correspondingly, as is common for this paradigm (e.g., Huettig and Altmann, 2005). Two epochs of the timeline are of particular interest. One one hand, expectations based on presentation frequency may be driven by information available from the word stem, and such an effect can be expected to be detectable in the time-window defined from 200 – 600 ms after onset of the target word, i.e., corresponding to the time between the onset of the word stem and the onset of the suffix. On the other hand, expectations may also be triggered while processing the suffix (e.g., for a given word stem, one suffix may be expected while the other is unexpected), and the second time-window of interest is therefore defined as the range from 600–1,000 ms after the onset of the word.

**Figure 5:**
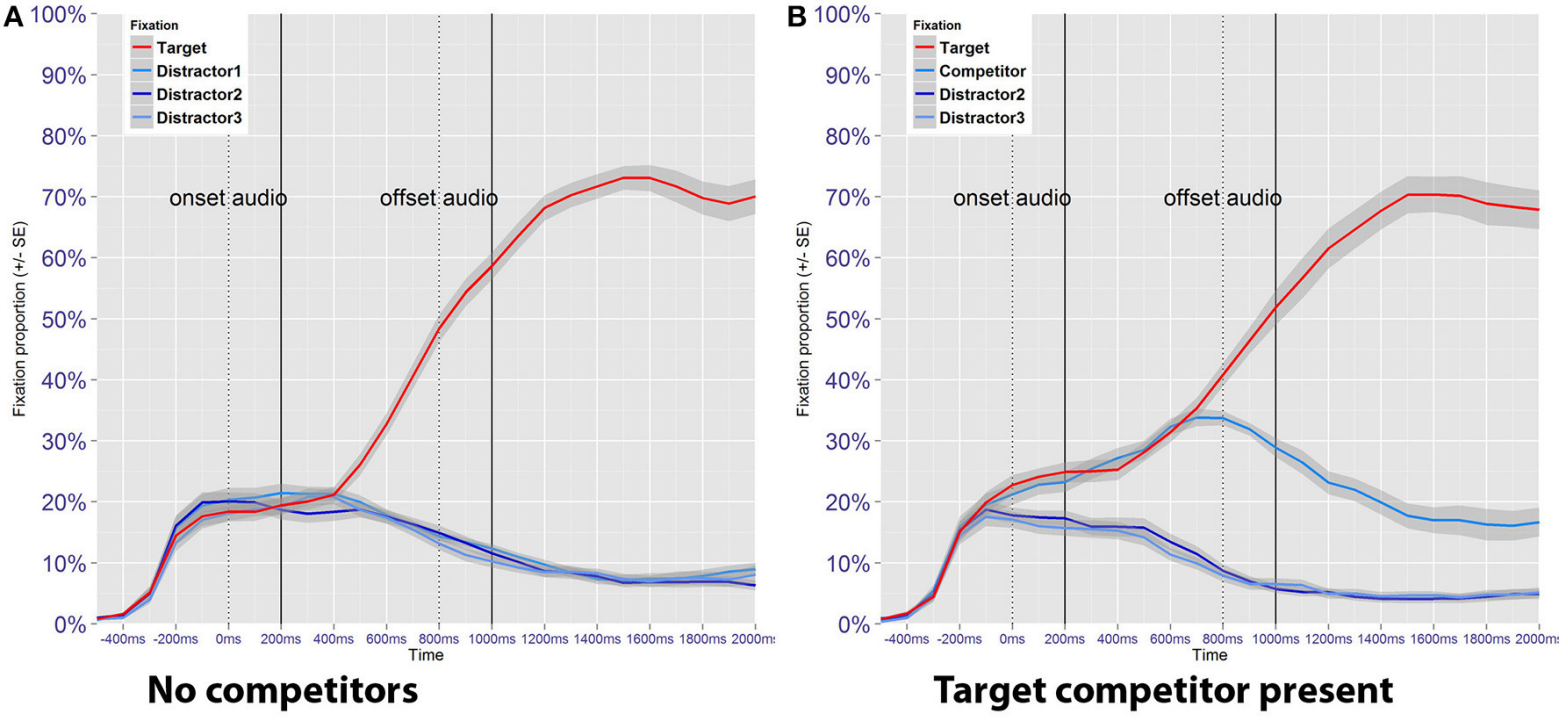
Proportion of fixations toward the target image as a function of time, plotted separately for no competitor trials **(A)** and target competitor trials **(B)**. Blue lines represent fixation proportions toward the three distractor images, which in **(B)** features a target competitor. Dotted lines represent approximate acoustic onsets and offsets for the auditory stimuli, while the solid lines include a 200 ms shift to account for the temporal lag between language processing and eye movement execution.

For an initial exploration, the proportion of fixations toward target and distractor images was calculated in time bins of 100 ms, aggregated by subject and trial type (Figure 5). Correct and incorrect responses are included in order to reflect overall timing of stimulus events and to capture global patterns in the data. When no image pairs are present (Figure 5A), participants are equally likely to fixate either of the four images until 400 ms after the acoustic onset of the target word. From this timepoint onwards incoming auditory information is used incrementally to identify the correct image, as reflected in the increased number of fixations toward the target image. Also in line with the expectations, Figure 5B shows that fixation proportions toward the target image and the target competitor do not bifurcate until disambiguating information from the suffix becomes available. This happens approximately 600–700 ms after the acoustic onset (only after information from the first 400–500 ms of the word has been processed) which coincides with the onset of the suffix. An additional pattern apparent in Figure 5B is that fixations to the image pair is higher even before auditory information is available. This pattern was also found in distractor competitor trials (not shown in the figure), and indicates that the mere presence of an image pair attracted participants' attention.

The analysis presented in the following sections is conducted on the two time windows as previously defined, after the removal of trials with incorrect responses. Conducting separate analyses on the different time windows also allows time to be modeled as a linear predictor, which facilitates model specification, estimation and interpretation. The analysis of trials without an image pair is followed by a separate analysis of target competitor trials. These trial types were analyzed separately because the initial exploration revealed that the overall gaze patterns differ, and because target competitor trials allow for a direct comparison between low and high frequency items within the same display.

#### 3.4.1. Trials without an image pair (no competitor trials)

**Figure 6:**
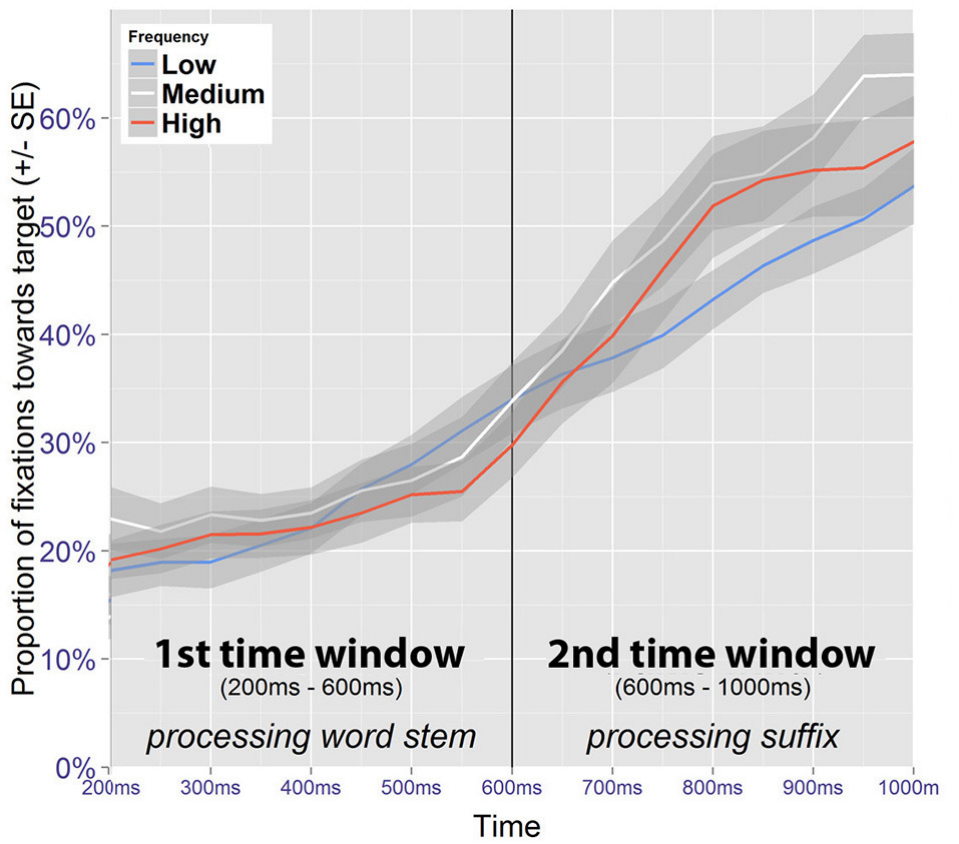
Fixation proportions toward the target according to its frequency (no competitor trials).

When a model includes time as a fixed effect, time is recalculated to range from 0 at the beginning to 1 at the end of the time window. Thus, the intercept represents model estimates at the onset of the time window. In order to obtain estimates at the end of the time window under investigation, time is recentered to range from -1 to 0, prior to reestimating the same model. As these steps do not affect model fit, they are not explicitly reported. In the text, the relevant estimates derived from these models are reported in percentages. Figure 6 presents fixation proportions toward the target figure according to its presentation frequency in relation to the two time windows of interest.

##### 3.4.1.1. Word stem

For the gaze patterns in the time window corresponding to the processing of the word stem (200–600 ms after auditory onset), only target fixations are included in the analysis. To retain maximal temporal resolution, unaggregated data were used.

The best fitting model contains fixed effects of time and frequency along their interaction term (Table 3). At the beginning of the time-window, the fixation proportion toward low frequency targets is estimated at 13.6% (95% CI [10.3, 17,8]). This estimate is significantly higher for medium frequency targets (18.6%, 95% CI [14.5, 22.6], *z* = 2.47, *p* < 0.05). For high frequency targets, the estimate is also higher, but this difference is not significant (15.9%, 95% CI [12.1, 20.6], *z* = 1.14, *p* = 0.256). At the end of the time window, the fixation proportion toward low frequency targets is estimated at 28.6% (95% CI [22.2, 36.0]). By comparison, medium frequency targets have a fixation proportion of 25.5% (95% CI [20.8, 30.8]), which is not significantly different (*z* = −1.17, *p* = 0.24). For high frequency items, the estimate is 23.0% (95% CI [18.0, 28.8]), and this is marginally significant (*z* = −1.89, *p* = 0.059).

**Table 3:** Model estimates, first time window.

	**Estimate**	**S.E**	***z*-value**	**Pr(>t)**
(Intercept)	−1.848	0.164	−11.28	<0.001
**FREQUENCY**				
Medium	0.343	0.139	2.47	<0.05
High	0.182	0.160	1.14	0.256
**TIME**	0.933	0.205	4.56	<0.001
Time^*^Medium	−0.499	0.142	−3.51	<0.001
Time^*^High	−0.477	0.146	−3.27	<0.01

Although these results suggest frequency-based information to be effective during online processing of the stem, their interpretation is not straight forward. Crucially, the increased amount of fixations is observed at the beginning of the time window, and is therefore likely to be a spurious effect, as a language driven effect would be expected to increase (or at least to be sustained) within the time window.

##### 3.4.1.2. Suffix

To investigate possible frequency effects coinciding with the processing of the suffix, fixations toward the target image were analyzed in the time window ranging from 600–1,000 ms after the onset of the target word.

Significant effects were found for frequency and time, along their interaction (Table 4). Low frequency images received 31% of the fixations (95% CI [26, 36]) at the beginning of the time window. This is not significantly different from the estimate obtained for medium frequency images (32%, 95% CI [26, 40], *z* = 0.43, *p* = 0.668), nor for high frequency images (30%, 95% CI [24, 38], *z* = −0.18, *p* = 0.861). In contrast, at the end of the time window the fixation proportion for low frequency images is estimated at 50% (95% CI [44, 56]). This estimate is significantly higher for medium frequency images (63%, 95% CI [56, 70], *z* = 3.43, *p* < 0.001), and also for high frequency images (61%, 95% CI [53, 69], *z* = 2.6, *p* < 0.01).

**Table 4:** Model estimates, second time window.

	**Estimate**	**S.E**	***z*-value**	**Pr(>t)**
(Intercept)	−0.804	0.125	−6.42	<0.001
**FREQUENCY**				
Medium	0.068	0.158	0.43	0.668
High	−0.031	0.175	−0.18	0.861
**TIME**	0.813	0.149	5.47	<0.001
Time^*^Medium	0.472	0.122	3.87	<0.001
Time^*^High	0.485	0.123	3.93	<0.001

The results suggest a processing disadvantage for stimuli that were inconsistent with the induced expectations. That the effect of frequency is not manifest at the beginning of the time window, but rather emerges within it, further suggests that this effect is at least partially driven by the processing of the suffix, as opposed to being a continuation of the potentially spurious effect observed in the preceding time window. A possible explanation would be that participants first used auditory information from the stem to identify the correct figure, but were expecting a different suffix, and therefore became uncertain at this timepoint.

#### 3.4.2. Trials featuring a target competitor (target competitor trials)

The presence of an image pair in a singular display offers the opportunity to investigate fixations toward low and high frequency images as paired observations. If an image pair for which a bias has been introduced is being fixated, the participant is either looking at the figure consistent with the induced expectation, or she is looking at the figure that is inconsistent with this expectation. Crucially, participants are also either looking at the male or the female member of the pair, and may show systematic choices in which of the two is fixated first. Acknowledging these dependencies, only fixations toward the image pair were analyzed. The adequacy of this approach is supported by the fact that the presence of an image pair generally attracts participants' attention (Figure 5), which results in the number of observations being higher than for no competitor trials.

**Figure 7:**
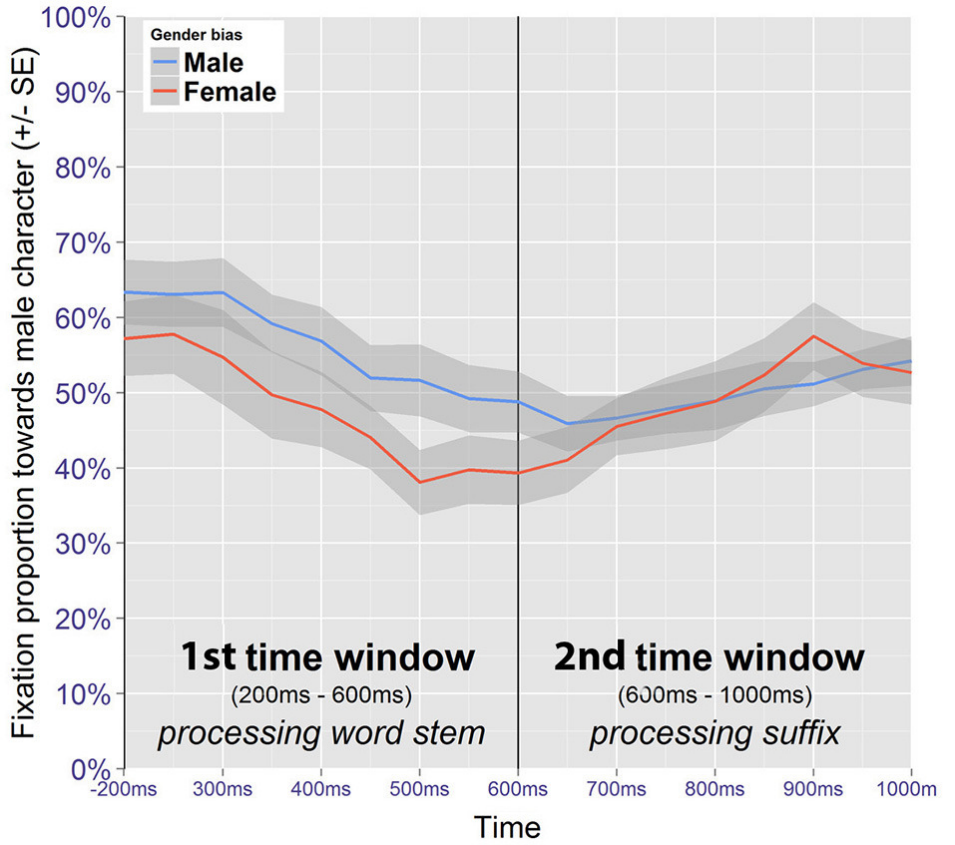
Gazedata from target competitor trials (image pair fixations only). Mean proportions represent fixations toward the male member of the pair.

Data were recoded to define fixations toward the male image as the dependent variable, and the analyses investigated the effects of time on the one hand and of the induced gender bias on the other. For trials where the male figure had a high presentation frequency during training, the gender bias is referred to as being male, while for trials where the male figure had had a low presentation frequency during training, the gender bias is referred to as being female. Figure 7 shows the fixation proportions toward the male member of the pair for the two time windows of interest, according to the induced gender bias. The global pattern suggests that participants tended to fixate the male figure first.

##### 3.4.2.1. Word stem

The first time window (200–600 ms after onset of the word) was analyzed for trials featuring a target competitor. Only correct trials where a bias had been introduced were included in the analysis. The best fitting model contains fixed effects for time and bias, along their interaction (Table 5). When there was a male bias, male images received 65.3% of the fixations at the beginning of the time window. When there was a female bias, fixations toward male images was lower at 59.9%, but the difference was not significant (*z* = 1.11, *p* = 0.267). At the end of the time window, the fixation proportion toward the male image had dropped to 47.5% when there was a male bias, and to 31.9% when there was a female bias. This difference was significant (*z* = 3.22, *p* < 0.01). Thus, despite the initial and bias-independent preference for fixating the male image at the beginning of the time window, when there was a female bias, there was also a clear preference for fixating the female image at the end of the time window. This suggests that probabilistic information available from the word stem was indeed used to predict which of the two images was going to be the referent.

**Table 5:** Model estimates, first time window.

	**Estimate**	**S.E**		***z*-value**	**Pr(>z)**
(Intercept)	0.633	0.205		3.09	<0.01
**GENDER BIAS**					
Female	−0.232	0.209		−1.11	0.267
**TIME**	−0.732	0.248		−2.95	<0.01
Time^*^Female	−0.428	0.182		−2.36	<0.05

##### 3.4.2.2. Suffix

The same analysis strategy was used on the time window that coincides with the processing of the suffix (600–1,000 ms after the onset).

Male images receive 44.1% of the fixations at the beginning of the time window when the bias is toward the male image (Table 6). When the bias is toward the female image, the estimate is lower at 37.8%, but not significantly different (*z* = -1.30, *p* = 0.194). At the end of the time window, the fixation proportion toward the male image has increased to 54.1% when there is a male bias and to 59.4% when there is a female bias, but again the difference is not significant (*z* = 0.197, *p* = 0.273).

**Table 6:** Model estimates, second time window.

	**Estimate**	**S.E**		***z*-value**	**Pr(>z)**
(Intercept)	−0.239	0.167		−1.43	0.153
**GENDER BIAS**					
Female	−0.260	0.200		−1.30	0.194
**TIME**	0.403	0.195		2.07	<0.05
Time^*^Female	0.476	0.155		3.07	<0.01

The gaze patterns show that at the beginning of the second time window, male figures receive more attention when there is a male bias, compared to when there is a female bias, and that this difference is attenuated over time. Nevertheless, this difference does not reach significance. Regardless of its origin, the attenuation of the effect within the time window suggests that disambiguating gender information from the suffix was indeed used to identify the correct image.

In summary, the patterns observed in the preceding analyses indicate that frequency information affects online processing. When no competitor was present, a preference for looking at the high frequency image was detected during the processing of the suffix. When a target competitor was present, the preference for the high frequency image was detected during processing of the stem.

## 4. General discussion

The present study addresses the emergence of experience-based gender expectations in an implicit learning situation, using an artificial language paradigm. The results show that during the acquisition of a miniature artificial language consisting of gender-marked pseudowords and associated visual referents, participants track the frequency of exposure to male vs. female realizations of words and referents, and thereby build up gender expectations inherent to the new representations. These expectations surface during subsequent processing, even if the gender-coding system underlying the materials was acquired implicitly. On a global level, the results of the current experiment replicate findings reported in an explicit learning scenario (Öttl and Behne, 2016). Whereas accuracy and response time data to some extent indicate that similar representations were acquired in the current and the replicated experiment, suggesting that experience-based expectations are robust against manner of acquisition, the investigation of gender coding errors and eyetracking data reveal a slightly more complex picture.

In terms of accuracy during acquisition, participants started off at 25% correct in the pre-test. Lacking explicit knowledge about the gender-coding system underlying the material, the four available candidate referents were equally plausible, and therefore 25% correct mirrors chance level performance. By the third training block, performance approaches ceiling, suggesting that the items have been successfully acquired. However, bearing in mind that gender information was redundant during the training blocks, as targets could be identified by the wordstems alone, the high accuracy at this stage likely overestimates participants' knowledge of the gender-coding system. Evidence that knowledge about the gender coding is indeed lower than the performance in the training blocks suggests can be found in the results from the post-test, where performance differs according to trial type. When an image pair is present (i.e., in target competitor and distractor competitor trials), performance is significantly worse relative to when it is not (no competitor trials). Accuracy being lower when an image pair is present suggests that the gender coding was at least to some extent problematic for the participants. At the same time, the fact that performance was lower both in target competitor trials and in distractor competitor trials indicates that it is not necessarily due to gender coding alone, since in the distractor competitor trials the drop in performance can only be attributed to the visual presence of an image pair among the distractors. The mere presence of an image pair in the visual display is therefore also likely to be a contributing factor to the lower performance in target competitor trials. The difference between target competitor and distractor competitor trials (94.6% vs. 96.5% correct respectively) did not reach significance. However, bearing in mind that performance was at 98.4% correct in trials without a competitor, the contrast in performance strongly suggests that the gender coding required to resolve target competitor trials makes this trial type even more difficult.

Turning to the post-test response time data, which offer a window on the newly formed representations that goes beyond mere accuracy, shorter response times were found for both medium and high probability items relative to low probability items, indicating that experience-based gender expectations were successully induced. Recognizing a referent whose gender is consistent with an induced expectation (or, in the case of medium probability items, items for which no gender expectation has been induced) is quicker than recognizing one whose gender is inconsistent with the induced expectation. No indication was found that the facilitation could be gradual depending on the probability, as a facilitation of 94 ms was observed for medium frequency items and one of 72 ms for high frequency items, a difference that did not reach significance. Additional evidence that experience-based gender expectations affected online aspects of processing was found in the eyetracking data. Overall, the gaze patterns were consistent with the expectations for the visual world paradigm to the extent that with incoming auditory information, participants became increasingly more likely to fixate the target referent, indicating that the newly acquired lexicon was processed similarly to natural words (see e.g., Dahan et al., 2001). Gender expectations were found to surface during online processing of the words, with increased fixations toward target referents whose gender was consistent with an induced expectation relative to targets whose gender was inconsistent with an induced expectation. When a target competitor was present, this effect was observed during the processing of the stem, and it was attenuated during the processing of the suffix. Also in no competitor trials, this effect was observed, but only in the time window corresponding to the processing of the suffix.

Globally, the patterns outlined above are very similar to the patterns reported for an explicit learning scenario in Öttl and Behne (2016), with only a few exceptions. The most evident difference between results from the two experiments is to be found in the accuracy data. In both experiments, participants started at chance level performance. In the current experiment, chance level lies at 25% correct, as the participants had to select one out of four possible referents. In Öttl and Behne (2016) however, participants were explicitly instructed about the gender-coding system underlying the artificial language and were also aware of the visual gender cues, and therefore chance level performance in that experiment is at 50%, as the display always featured two male and two female characters. Though in both experiments performance approaches ceiling in the third training block, suggesting that the word-referent associations are mastered to a similar degree regardless of the mode of acquisition, an examination of the performance in the post-test reveals that this initial assessment is too superficial. While in an explicit learning situation, participants performed at ceiling regardless of trial type, the presence of an image pair led to a weakened performance in an implicit learning situation. Thus, even if performance is very high in both experiments, the gender coding system cannot be said to be perfectly mastered in the implicit learning situation employed in the current experiment. When it comes to the induced gender expectations as reflected in the response time data, the patterns found in the current experiment are very similar to those reported in Öttl and Behne (2016). Both in an implicit and in an explicit learning situation, a significant facilitation is found for both medium and high probability items relative to low probability items. Whereas Öttl and Behne (2016) find the facilitation to be by 27 and 96 ms for medium and high probability items respectively, such a gradual effect is not found in the present experiment. Nevertheless, the overall extent of the facilitation is highly consistent across the two experiments, approaching a maximum contrast of 100 ms. We do not have a plausible explanation for why the effect is not gradual in an implicit learning scenario, but acknowledge that in neither experiment was a significant difference found between medium and high probability items. Even if the gender expectations are not perfectly mirrored in the current replication experiment, it is noteworthy that they appear to a similar extent, particularly if seen in light of the general differences found for gender coding *per se*.

Additional evidence that an implicit learning scenario led to difficulties with gender coding relative to an explicit learning scenario can be found by comparing the response times according to trial type across the two experiments. In the current experiment, the presence of a distractor competitor led to a facilitation of 63 ms when compared to trials in which no competitors were present, which is similar to the facilitation of 56 ms reported for an explicit learning scenario (Öttl and Behne, 2016). This facilitation is likely due to the fact that the image pair can be quickly eliminated from consideration, reducing the number of available candidates. For target competitor trials on the other hand, the findings are more divergent, with the current experiment yielding a delay of 145 ms, compared to only 75 ms in Öttl and Behne (2016). As argued in Section 3.3, finding longer response times for target competitor trials must be partially attributed to the fact that auditory information from the suffix is required to resolve reference, and it is not clear whether the 75 ms delay reported in Öttl and Behne (2016) may be fully attributed to this aspect, or if it is also partly due to target competitor trial being experienced as more difficult as well. In either case, a longer delay for target competitor trials in the current experiment is consistent with the difficulties with gender coding observed in accuracy measures.

One limitation to the above comparison between the current and the replicated experiment is that the contrast between implicit and explicit learning is understood as a contrast between providing and not providing participants with knowledge about the gender coding system underlying the material to be acquired. In Öttl and Behne (2016), participants were shown an example of an image pair and informed which suffix encoded which gender. Distinguishing to which extent the differences in results can be attributed to visual vs. linguistic aspects of processing is therefore not possible. The overall gaze patterns are similar across the two studies, except for the gender expectations seeming to arise somewhat earlier in the current experiment. One possible explanation for this contrast is that it is driven primarily by differences in visual attention during the inspection of the figures. Support for this view can be found in the overall gaze patterns, where Öttl and Behne (2016) report that during the processing of the suffix in no competitor trials, participants were more likely to fixate the distractor of the same gender as the target than those of the opposite gender, an indication that the suffix was used actively to guide visual attention. A corresponding effect was not detected in the current experiment.

That experience based gender expectations seem at least to some extent to be independent of overt attention to gender information provides support for the finding that with real words, gender expectations are hard or difficult to suppress (Oakhill et al., 2005). In the current experiment, participants were not instructed or in any way encouraged to pay attention to gender information, and results show that the gender-coding system was at least to some extent difficult to acquire. Nevertheless, the sensitivity to probabilistic gender information seems to come very close to that observed in an explicit learning situation. Though for real words, the timescale of acquisition and the complexity of referents and learning contexts are undeniably of a different scale than the simulations presented here, a central implication of these findings is that experience-based gender expectations reflect statistical regularities in the input, regardless of whether the categories these regularities belong to are highlighted or not.

## 5. Conclusion

By replicating an experiment investigating the formation of frequency based expectations in an artificial language, while changing the learning situation from an explicit to an implicit one, the present experiment contributes to understanding the impact different modes of acquisition may have on the formation of new representations. Finding that the acquisition of a gender coding system proceeds less successfully in an implicit learning scenario than in an explicit learning scenario may not be surprising in its own right, since in the latter case what it to be learned is already given away. More importantly, the finding that frequency based expectations seem to surface to similar extents in both learning scenarios indicates that certain aspects of newly formed representations are robust against manner of acquisition. This finding has implications not only for understanding differences between different modes of acquisition, but also for understanding the cognitive representation of gender information, particularly why gender expectations are activated, even in cases where they are not relevant for discourse coherence.

## Ethics statement

This study did not involve the collection or storage of any personal data, and did therefore not require clearance by an ethical committee. All subjects gave their written informed consent, and were free to withdraw at any stage during the study. The protocol was approved by NSD (Norwegian Centre for Research Data).

## Author contributions

Both authors contributed extensively to the work presented in this paper. AÖ and DB jointly conceived of the study and sketched the design. AÖ carried out much of the theoretical and practical implementation of the project, and drafted the full paper. DB supervised all stages of the project. Both authors discussed the results and implications and contributed to the manuscript at all stages.

### Conflict of interest statement

The authors declare that the research was conducted in the absence of any commercial or financial relationships that could be construed as a potential conflict of interest.
