# Supplementary material for: Assessing the Formation of Experience-Based Gender Expectations in an Implicit Learning Scenario
Source: Front Psychol. 2017 Sep 7;8:1485. doi: 10.3389/fpsyg.2017.01485 (PMC5594219; doi:10.3389/fpsyg.2017.01485)
Supplement: Supplementary file 1 [file Table1.pdf]

Supplementary Table 1. Model comparisons for response time data.

Model	Comparison	Fixed effects	Log lik	df	χ^2	p(>χ^2)
A	Null	Trial Type	-585	2	17.95	<0.001
B	A	Trial Type + Frequency	-580	2	10.03	<0.01
C	B	Trial Type * Frequency	-578	4	5.27	0.261

Supplementary Table 2. Model comparisons for gaze data. First time window, no competitor trials.

Model	Comparison	Fixed effects	Log lik	df	χ^2	p(>χ^2)
A	Null	Time	-11100	1	8.25	<0.01
B	A	Time + Frequency	-11100	2	1.27	0.531
C	B	Time * Frequency	-11092	4	16.52	<0.01

Supplementary Table 3. Model comparisons for gaze data. Second time window, no competitor trials.

Model	Comparison	Fixed effects	Log lik	df	χ^2	p(>χ^2)
A	Null	Time	-14068	1	28.18	<0.001
B	A	Time + Frequency	-14066	2	4.38	0.110
C	B	Time * Frequency	-14056	4	24.7	<0.001

Supplementary Table 4. Model comparisons for gaze data. First time window, target competitor trials.

Model	Comparison	Fixed effects	Log lik	df	χ^2	p(>χ^2)
A	Null	Time	-4486	1	7.25	<0.01
B	A	Time + Gender Bias	-4484	1	5.46	<0.05
C	B	Time * Gender Bias	-4481	1	5.46	<0.05

Supplementary Table 5. Model comparisons for gaze data. Second time window, target competitor trials.

Model	Comparison	Fixed effects	Log lik	df	χ^2	p(>χ^2)
A	Null	Time	-6024	1	9.57	<0.01
B	A	Time + Gender Bias	-6024	1	0.00	0.953
C	B	Time * Gender Bias	-6019	1	9.38	<0.01
